# Extracellular Vesicles Derived from Gut Microbiota, Especially *Akkermansia muciniphila*, Protect the Progression of Dextran Sulfate Sodium-Induced Colitis

**DOI:** 10.1371/journal.pone.0076520

**Published:** 2013-10-24

**Authors:** Chil-sung Kang, Mingi Ban, Eun-Jeong Choi, Hyung-Geun Moon, Jun-Sung Jeon, Dae-Kyum Kim, Soo-Kyung Park, Seong Gyu Jeon, Tae-Young Roh, Seung-Jae Myung, Yong Song Gho, Jae Gyu Kim, Yoon-Keun Kim

**Affiliations:** 1 Department of Life Sciences, Pohang University of Science and Technology (POSTECH), Pohang, Republic of Korea; 2 Department of Gastroenterology, Asan Medical Center, University of Ulsan College of Medicine, Seoul, Republic of Korea; 3 Department of Medicine, Chung-Ang University College of Medicine, Seoul, Republic of Korea; Universidad Andres Bello, Chile

## Abstract

Gut microbiota play an important part in the pathogenesis of mucosal inflammation, such as inflammatory bowel disease (IBD). However, owing to the complexity of the gut microbiota, our understanding of the roles of commensal and pathogenic bacteria in the maintenance of immune homeostasis in the gut is evolving only slowly. Here, we evaluated the role of gut microbiota and their secreting extracellular vesicles (EV) in the development of mucosal inflammation in the gut. Experimental IBD model was established by oral application of dextran sulfate sodium (DSS) to C57BL/6 mice. The composition of gut microbiota and bacteria-derived EV in stools was evaluated by metagenome sequencing using bacterial common primer of 16S rDNA. Metagenomics in the IBD mouse model showed that the change in stool EV composition was more drastic, compared to the change of bacterial composition. Oral DSS application decreased the composition of EV from *Akkermansia muciniphila* and *Bacteroides acidifaciens* in stools, whereas increased EV from TM7 phylum, especially from species *DQ777900_s* and *AJ400239_s*. *In vitro* pretreatment of *A. muciniphila*-derived EV ameliorated the production of a pro-inflammatory cytokine IL-6 from colon epithelial cells induced by *Escherichia coli* EV. Additionally, oral application of *A. muciniphila* EV also protected DSS-induced IBD phenotypes, such as body weight loss, colon length, and inflammatory cell infiltration of colon wall. Our data provides insight into the role of gut microbiota-derived EV in regulation of intestinal immunity and homeostasis, and *A. muciniphila*-derived EV have protective effects in the development of DSS-induced colitis.

## Introduction

Inflammatory bowel disease (IBD) is a disease referring to the chronic or recurring immune response and inflammation of the intestines [1]. IBD comprises of two major types Crohn's disease and ulcerative colitis (UC). The cause of IBD has been elusive until recent evidence gave substantial evidence regarding the relationship between gut microbiota and the regulation of intestinal immunity [2]. Currently, the pathogenesis of IBD is attributed to an inappropriate and continuing immune response to normal commensal bacteria in genetically susceptible individuals [3]. Microbiota, a diverse collection of microorganisms, appears to be a source of antigens that the immune system responds to thus inducing inflammation [4]. The specific antigenic properties of microbiota and the exact mechanisms involved have yet to be identified.

In normal individuals, gut microbiota and host create a symbiotic relationship. The intestine is an open ecological system that is constantly exposed to 500 to 1,000 species of commensal bacteria [5]. It provides a suitable environment that accommodates many flourishing gut microbiota communities of approximately 10^14^ organisms [6]. In return the microbiota acts as moderators of a healthy epithelial gut barrier by participating in several functions, including metabolic activities involved in salvaging energy and absorbing nutrients, trophic effects on the intestinal epithelial, promotion of gut maturation and integrity, maintenance of intestinal immune homeostasis, and defense against pathogenic bacteria [7]. Through a comprehensive culture-independent metagenomic analysis, increasing evidence has been found that there are significant differences in the composition of microbiota in IBD patients and healthy individuals [8]. Further investigation may present clues to causative and/or protective features of microbiota in relation to IBD.

A key part to the puzzle may lie in the mechanisms of crosstalk between microbiota and host that are involved in both health and disease. Bacteria produce extracellular vesicles (EV), including gut microbiota [9]. Communication of Gram-negative bacteria is commonly believed to occur through secretion of soluble mediators and EV, also called by outer membrane vesicles (OMV) [10]. Recent evidence also supports Gram-positive bacteria to secrete EV similar to Gram-negative bacteria [11]. Collectively, EV from both Gram-positive and Gram-negative bacteria may have a dualistic character regarding the host, some proving to be beneficial while others detrimental. Accumulating evidence gives support to the hypothesis that EV are involved in the mechanisms of immunity and disease.

To our knowledge, studies on stool-derived EV have not been reported yet. Moreover, there are no tracing data using bacterial metagenomics. In the present study, we hypothesized that the gut microbiota-derived EV are the key in modulating intestinal homeostasis and dysregulation. Here, we provide evidence that shows microbiota-derived EV to play a pivotal role in the pathogenesis of IBD and microbiota-derived EV is an important mediator in the maintenance of gut homeostasis.

## Results

### Characterization of stool EV from a dextran sulfate sodium (DSS) colitis mouse model

**Figure 1:**
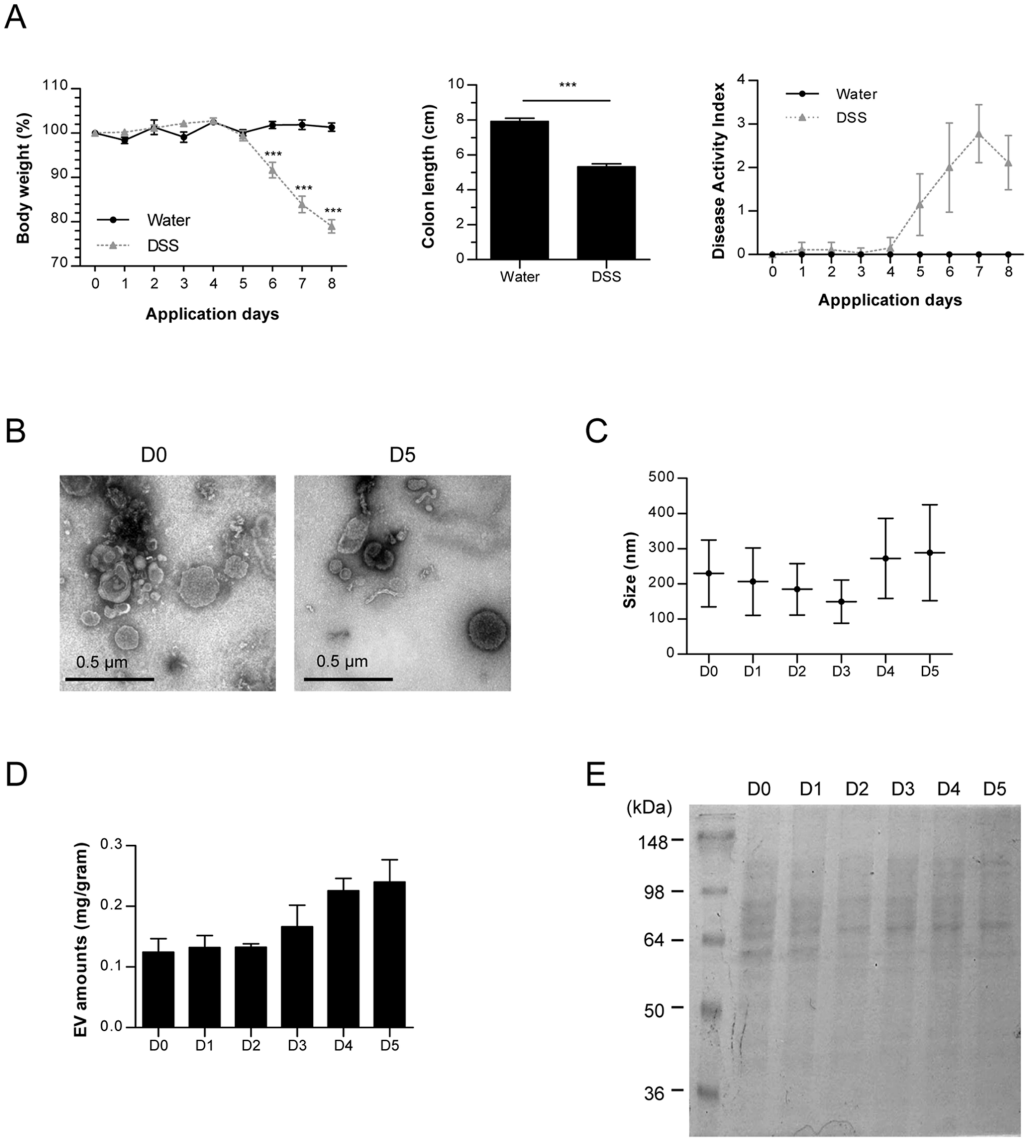
Characterization of stool EV from a DSS-induced colitis mouse model. For all figures, C57BL/6 mice (each group = 5) were ingested by water or 3% DSS. (A) Body weight changes (%, left panel), colon length (middle panel), and disease activity index including body weight, colon length, diarrhea, and stool blood (right panel). Water: water-administered group; DSS: 3% DSS-administered group; *, p<0.05; ***, p<0.001. (B) TEM images (×80 k) of EV isolated from stools on days 0 and 5. D0; stools before DSS application; D5: stools 5 days after 3% DSS application. (C) EV sizes (d.nm) measured by nanoparticle tracking analysis (NTA) on day 0 through day 5. (D) EV extraction amounts (protein concentration per gram stool) from stools D0 – D5. (E) Protein profiles in EV (D0 – D5) through SDS-PAGE by using coomassie brilliant blue G250 dye.

Compared to other strains, C57BL/6 strain of mice shows a higher susceptibility to DSS-induced colitis [12]. We administered 3% DSS solution to female C57BL/6 mice for a period of 5 days, and then evaluated disease phenotypes (**Fig. 1A**). Starting from the 6th day, mice treated with DSS began to lose weight rapidly so that by day 8, the DSS treated mice showed an approximate 20% loss of their initial body weight; the control mice showed no significant change in weight. Compared to the average control-group colon length of 8 cm, the average colon length of the 3% DSS group, approximately 5 cm, was significantly shorter. Also, disease activity index (DAI) score of the DSS group began to increase from day 4 and rose to value of 3 by day 6. Collectively, effective induction of colitis was achieved by administration of 3% DSS solution to female C57BL/6 mice.

For the characterization of the gut microbe-derived fecal EV, the EV collected daily from day 0 to day 5 were measured using transmission electron microscopy (TEM) and nanoparticle tracking analysis (NTA). TEM demonstrated the vesicular shape of the samples (**Fig. 1B**). NTA showed that fecal EV constituted from various origins, which explain the slight variation of average size (**Fig. 1C**). Average protein yield and the protein pattern of stool-derived EV were analyzed for the protein composition. As the days progress from D1 to D5, there was an increase of stool-derived EV protein yield (**Fig. 1D**) and protein bands in SDS-PAGE changed (**Fig. 1E**) indicating a change in composition of the fecal EV.

### The proportion of bacteria and bacteria-derived EV in stools

**Figure 2:**
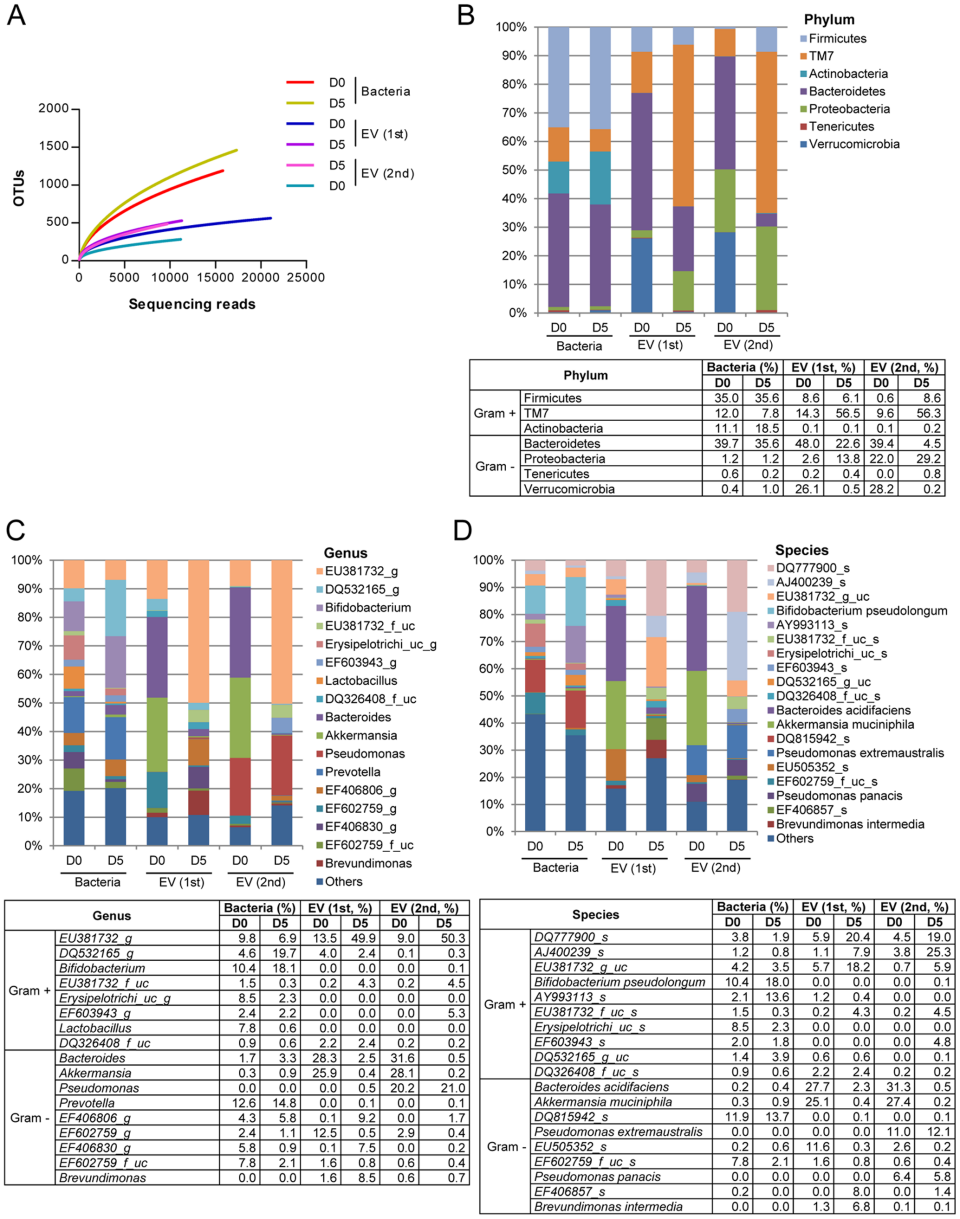
The composition of bacterial and bacteria-derived EV in stools following 3% DSS administration. For all figures, stool bacteria and EV were isolated from mice before (D0) and 5 days (D5) after 3% DSS oral administration (each group = 5). As for EV metagenomics, two independent experiments were performed. (A) Operational taxonomic units (OTUs) using Roche 454 GS FLX Titanium. For figures (B)–(D), the proportion of stool bacteria and bacterial EV is displayed at the phylum (B), genus (C), and species (D) levels. As for genus and species, the proportion less than 1% occupancy is noted as others.

Metagenomic analysis was used to evaluate the proportion of bacteria and bacteria-derived EV in large intestines. Stool samples were used to indirectly evaluate the proportion of bacteria and bacteria-derived EV in large intestines. Since fecal EV comprise of both host and bacteria derived EV, we amplified the EV using indigenous 16S rDNA to exclude host cell-derived EV. Then, the bacteria and bacteria-derived EV were assigned operational taxonomic unit (OUT) using the amplified 16S rDNA. Over a 1000 bacterial OTU were detected; however, EV OTU stayed well below 500 (**Fig. 2A**). This finding suggests that the intestines are host to a great diversity of bacteria but not all these bacteria produce EV, consequently bacteria-derived EV in the stools yielding a lower diversity than its source bacteria.

The composition of fecal bacteria and EV collected on Day 0 and Day 5 were compared at taxonomic levels. In the phylum level (**Fig. 2B**), there was a minor change in bacterial composition, reflected by 16S rDNA sequencing. Actinobacteria increased from 11.1% to 18.5%, Bacteroidetes decreased from 39.7% to 35.6%, TM7 decreased from 12.0% to 7.8%, and Firmicutes, Proteobacteria, Tenericutes, Verrucomicrobia stayed relatively the same. Interestingly, it was noted that compared to the slight shifts of bacterial composition, stool-derived EV 16S rDNA showed more drastic changes. Bacteroidetes decreased from 48.0% to 22.6% in the first set and 39.4% to 4.5% in the second set; Verrucomicrobia 26.1% to 0.5% and 28.2% to 0.2%. TM7 increased from 14.3%to 56.5% in the first set and 9.6% to 56.3% in the second set. The following showed less dramatic changes compared to the above: Firmicutes, 8.6% to 6.1% and 0.6% to 8.6%; Proteobacteria 2.6% to 13.8% and 22.0% to 29.2%; Tenericutes 0.2% to 0.4% and 0.0% to 0.8%. Actinobacteria EV showed almost no change. Even though bacteria composition changed with induction of colitis, in comparison the composition of fecal EV showed a more drastic change.

In the level of genus and species, similar to the phylum level, although bacteria showed shifts in composition, the change in EV composition was more drastic (**Fig. 2C and 2D**). For the level of genus and species, the vast diversity made it difficult to indicate all the genus and species thus there was a cut-off of 1% bacterial occupancy. In the genus level (**Fig. 2C**), bacterial composition that increased in IBD included *DQ532165_g, Bifidobacterium, Bacteroides, Akkermansia, Prevotella*, and *EF406806_g*. In contrast, *EU381732_g, EU381732_f_uc, Erysipelotrichi_uc_g, EF603943_g, Lactobacillus, DQ326408_f_uc, EF602759_g, EF406830_g*, and *EF602759_f_uc* were decreased in the disease state. As for the composition of bacteria-derived EV, DSS increased *EU381732_g, EU381732_f_uc, EF406830_g,* and *Brevundimonas* in one set; *EU381732_g, EU381732_f_uc*, and *EF406806_g* in another set. In contrast, DSS decreased EV from *Bacteroides, Akkermansia*, and *EF602759_g* in both sets. In terms of species level (**Fig. 2D**), DSS increased bacterial composition of *Bifidobacterium pseudolongum, AY993113_s, DQ532165_g_uc*, and *DQ815942_s*; whereas decreased that of *DQ777900_s, EU381732_g_uc, Erysipelotrichi_uc_s*, and *EF602759_f_uc_s*. EV 16S rDNA that increased in stools included *DQ777900_s, AJ400239_s, EU381732_g_uc, EU381732_f_uc_s*, and *DQ532165_g* in both sets, and *Brevundimonas intermedia* in one set. In contrast, oral application of 3% DSS for 5 days decreased EV from *Bacteroides acidifaciens* (27.7% to 2.3% and 31.3% to 0.5%), *Akkermansia muciniphila* (25.1% to 0.4% and 27.4% to 0.2%), and *EU505352_s* (11.6% to 0.3% and 2.6% to 0.2%) in both sets,

### The proportion of bacteria and bacteria-derived EV in small intestinal fluids

**Figure 3:**
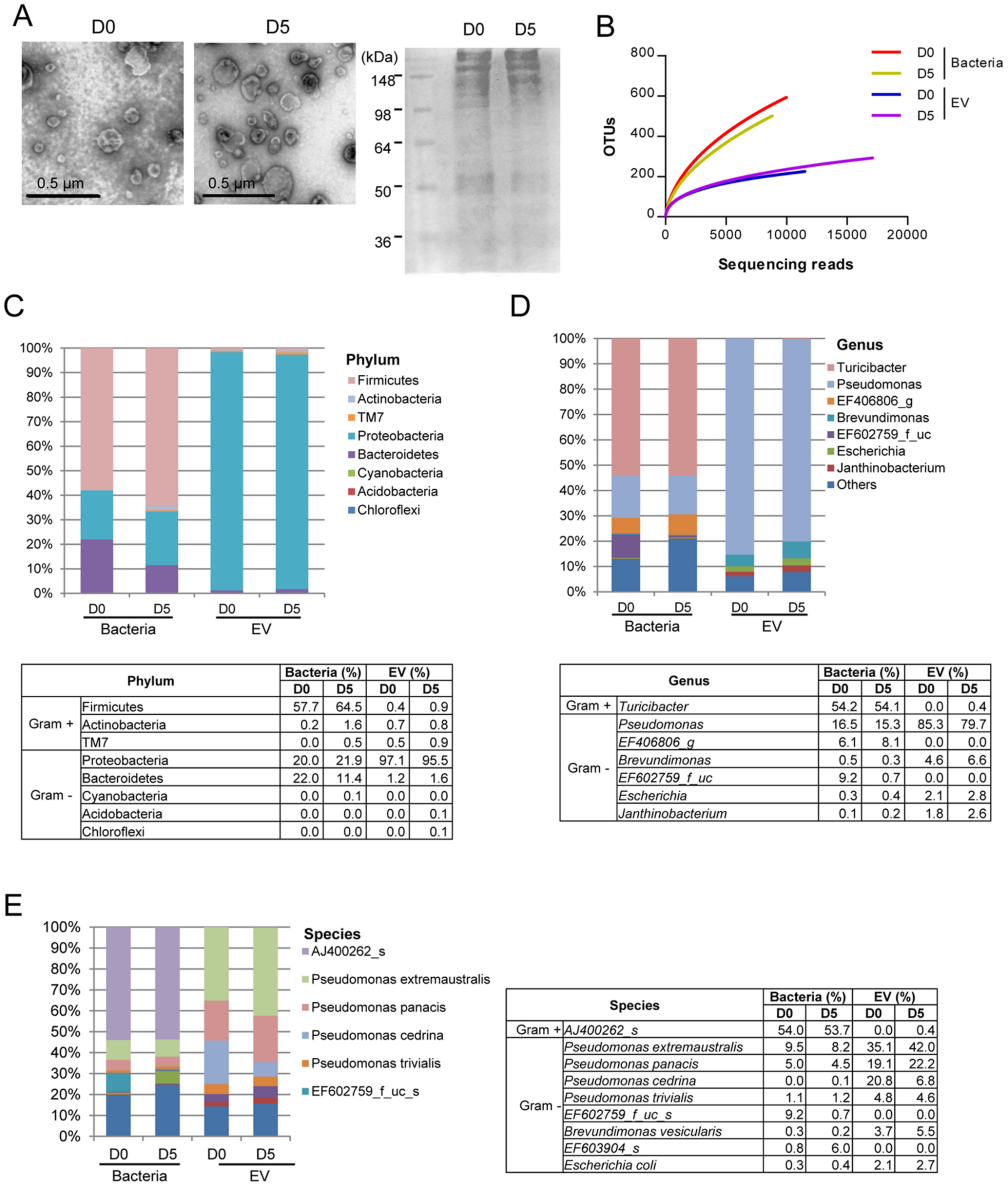
The composition of bacteria and bacteria-derived EV in small intestinal fluids following 3% DSS administration. For all figures, bacteria and EV were isolated from small intestinal fluids of mice before (D0) and 5 days (D5) after 3% DSS oral administration (each group = 5). (A) EV TEM images (×100 k, left panel) and protein profiles through SDS-PAGE by using coomassie brilliant blue G250 dye (right panel). (B) Operational taxonomic units (OTUs) of bacteria and bacterial EV using Roche 454 GS FLX Titanium. For figures (C)–(E), the proportion of bacteria and bacterial EV in small intestinal fluids is displayed at the phylum (C), genus (D), and species (E) levels. As for genus and species, the proportion less than 1% occupancy is noted as others.

For the characterization of EV in the small intestines, EV were isolated from the small intestinal fluids on days 0 and 5. TEM confirmed the vesicular shape of the EV, and protein bands in SDS-PAGE were similar between small intestinal EV on days 0 and 5 (**Fig. 3A**).

Metagenome analysis was used to evaluate the proportion of bacteria and bacteria-derived EV in small intestines. Over a 500 bacterial OTU were detected; however, EV OTU stayed below 300 (**Fig. 3B**). This finding suggests that the small intestines are also host to a great diversity of bacteria, but not all these bacteria produce EV. The composition of bacteria and EV in small intestines collected on Day 0 and Day 5 were compared at taxonomic levels. In the phylum level, there was a minor change in bacterial and EV compositions on days 0 and 5; the proportion of Firmicutes, Proteobacteria, and Bacteroides phyla on days 0 and 5 was 57.7% and 64.5%, 20.0% and 21.9%, and 22.0% and 11.4%, respectively; however, the phylum Proteobacteria was the main source of EV (97.1% and 95.5%) on days 0 and 5 (**Fig. 3C**).

In genus level (**Fig. 3D**), bacteria showed minor shifts in composition. Predominant bacteria on both days were *Turibacter* (54.2% and 54.1%), *Pseudomonas* (16.5% 15.3%), and *EF406806_g* (6.1% and 8.1%); however, *EF602759_f_uc* decreased in the disease state (9.2% to 0.7%). Similar to bacterial composition, the change in EV composition was minor; *Pseudomonas* was predominant (85.3% and 79.7%), and then *Brevundimonas* followed (4.6% and 6.6%) on days 0 and 5. In terms of species level (**Fig. 3E**), bacteria higher than a cut-off of 1% occupancy were *AJ400262_s* (54.0% and 53.7%), *Pseudomonas extremaustralis* (9.5% and 8.2*%), EF6.2759_f_uc_s* (9.2% and 0.7%), *Pseudomonas panacis* (5.0% and 4.5%), and *Pseudomonas trivialis* (1.1% and 1.2%) on days 0 and 5. As for EV composition, EV from *P. extremaustralis, P. cedrina, P. panacis, P. trivialis, Brevundimonas vesicularis*, and *Escherichia coli* occupied higher than 1% of total EV; among these, EV from *P. cedrina* decreased, whereas EV from *P. extremaustralis, P. panacis*, and *B. vesicularis* mildly increased in the disease state.

### Selection of candidate bacteria-derived EV based on metagenomics

**Figure 4:**
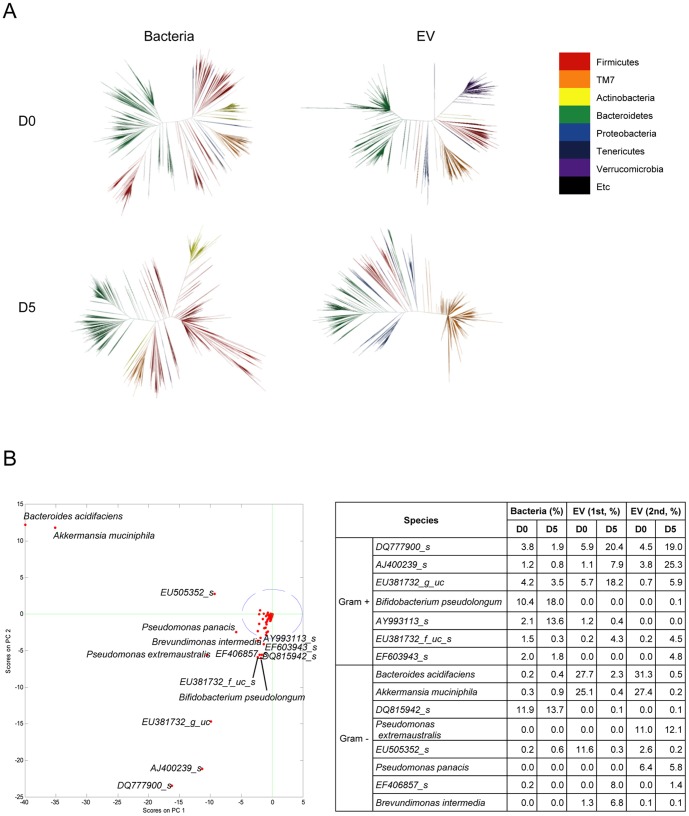
Change of stool bacteria and bacterial EV following 3% DSS administration. For all figures, stool bacteria and EV were isolated from mice before (D0) and 5 days (D5) after 3% DSS oral administration (each group = 5). As for EV metagenomics, two independent experiments were performed. (A) Phylogenetic trees of stool bacteria and bacterial EV. (B) Principal component analysis (PCA) of bacterial EV (left panel) and changes of candidate bacteria and bacterial EV following 3% DSS administration (right panel). Blue circle means 95% confidence interval, and green cross means the starting point to diverge each species.

In order to visualize the distribution of bacteria and bacteria-derived EV in stools, phylogenetic trees were drawn (**Fig. 4A**). The branches were divided according to the similarity of their sequences. The distribution and occupation of bacteria-derived 16S rDNA remained relatively unchanged. The level of EV-derived 16S rDNA showed greater differences, compared to bacterial 16S rDNA.

For statistical analysis of significant change in species composition, principal component analysis (PCA) was performed (**Fig. 4B**). At a confidence interval of 95%, any score outside the dotted line in the graph reflects statistical significance. 15 species were shown to have changed at a statistically significant level. Induction of colitis similar to UC caused a greater shift in composition of bacteria-derived EV in large intestines compared to the minor shifts in composition of bacteria suggesting a stronger association of IBD and EV from the gut microbiota. EV from 7 Gram-positive and 8 Gram-negative bacteria changed significantly in the disease state. Among these, EV from *B. acidifaciens* (set one: 27.7% to 2.3%; set two: 31.3%to 0.5%) and *A. muciniphila* (set one: 25.1% to 0.4%; set two 27.4% to 0.2%) decreased at prominent levels. In contrast, among EV from the TM7 phylum, EV derived from species *DQ777900_s* and *AJ400239_s* increased drastically. Bacteria-derived EV that increased as colitis progressed suggests a complementary relationship between the disease and EV. Bacteria-derived EV that had an inverse relationship with progression of colitis may suggest a protective nature of bacteria-derived EV in normal intestinal immunity. With the data we have presented so far, it's possible to conceive that in the progression of colitis, there is a breakdown in normal immunity maintained by *B. acidifaciens*-derived EV and *A. muciniphila*-derived EV.

### The preparation and characterization of *A. muciniphila*-derived EV

**Figure 5:**
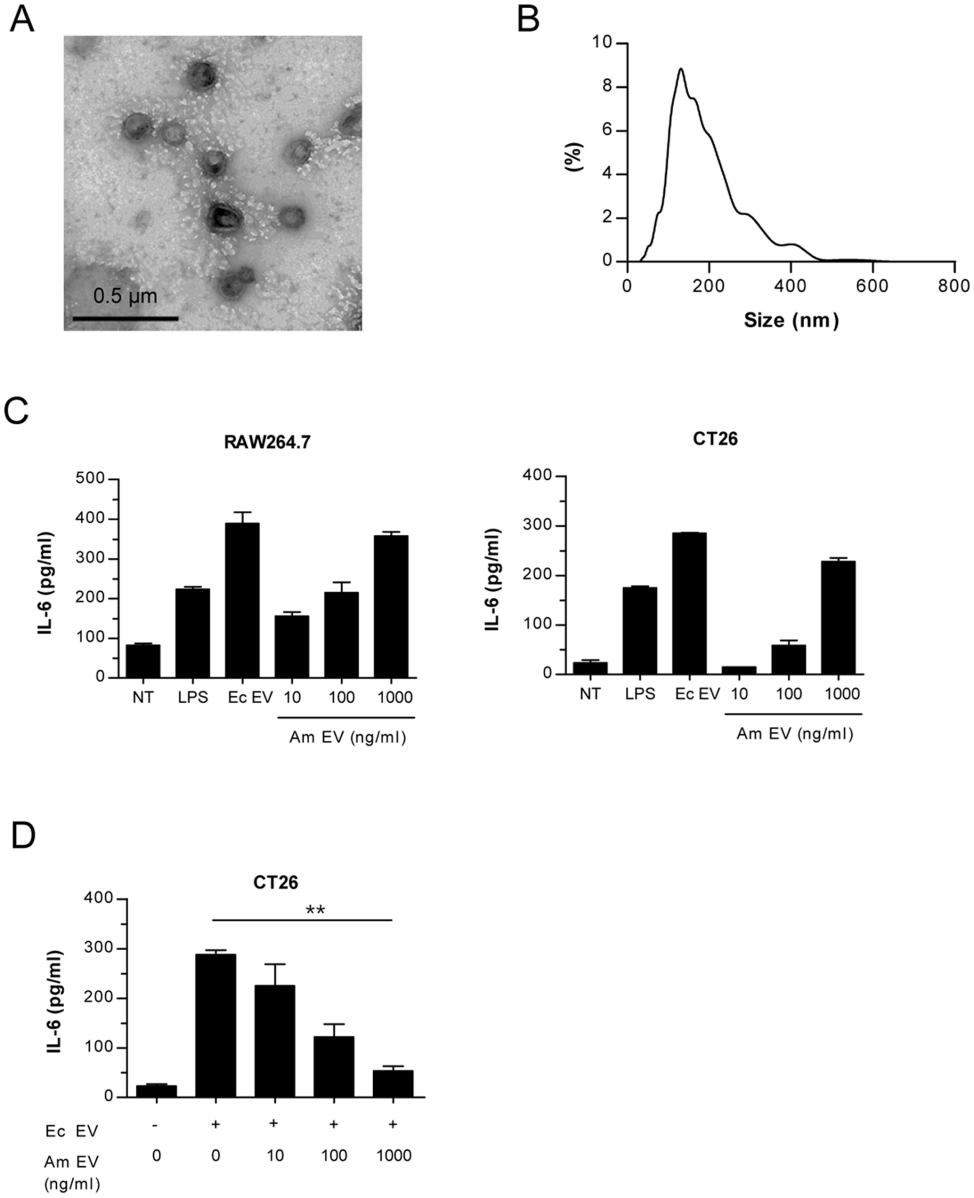
Characterization and immunogenicity of *A. muciniphila*-derived EV. (A) A TEM image (×100 k) showing a spherical shape of *A. muciniphila* EV. (B) Size (d.nm) of *A. muciniphila* EV measured by NTA. (C) Levels of a pro-inflammatory cytokine IL-6. IL-6 was measured 12 h after *A. muciniphila*-derived EV treated to peritoneal macrophage cell line (Raw264.7, left panel) and colon epithelial cell line (CT26, right panel). LPS: lipopolysaccharide, 75 ng/ml, Ec EV: *E. coli*-derived EV, 100 ng/ml; Am EV: *A. muciniphila*–derived EV. (D) *In vitro* anti-inflammatory effect of *A. muciniphila* EV. IL-6 levels were measured in supernatants of colon epithelial cells (CT26) after *A. muciniphila* EV were pre-treated to CT26 for 12 h and then 100 ng/ml of *E. coli* EV treated for 12 h. **, p<0.01 by ANOVA and test of linearity.

*A. muciniphila*-derived EV were chosen as our candidate in the next part of the experiment as it is possible to culture the species *in vitro*. *A. muciniphila* was cultured and then *A. muciniphila*-derived EV were extravagated. For characterization of the EV, the EV collected was evaluated using TEM (**Fig. 5A**) and NTA (**Fig. 5B**). TEM showed the vesicular shape of the EV, and average size measured using NTA was 198.13±86.76 nm. To examine immunogenicity, a murine peritoneal macrophage cell line (Raw 264.7) and gut epithelial cell line (CT26) were treated separately with media, LPS 75 ng/ml, *E. coli*-derived EV 100 ng/ml, and *A. muciniphila*-derived EV at concentrations of 10 ng/ml, 100 ng/ml, and 1 µg/ml (**Fig. 5C**). *A. muciniphila*-derived EV generated increasing IL-6 secretions in a dose-dependent manner. However, the pro-inflammatory effect of *E. coli*-derived EV was much higher than *A. muciniphila*-derived EV even at a greater concentration. This indicates a pro-inflammatory effect of *A. muciniphila*-derived EV; however it is weak in comparison to a pathogenic bacteria-EV like *E. coli*-derived EV thus reflecting a weaker potency of *A. muciniphila*-derived EV.

### The protective effect of *A. muciniphila*-derived EV in IBD pathogenesis

To elucidate the protective effects of *A. muciniphila*-derived EV, a colon epithelial cell line (CT26) were treated with *A. muciniphila*-derived EV at three different concentrations (10 ng/ml, 100 ng/ml, and 1 µg/ml) for 12 hours, as shown in **Fig. 5D**. Each of the pretreated cell line was then treated with 100 ng/ml of *E. coli*-derived EV. Cells treated only with *E. coli*-derived EV induced a much higher cytokine production compared to the cells pretreated with *A. muciniphila*-derived EV. As the concentration of pretreated *A. muciniphila*-derived EV increased, *E. coli* EV produced lower amounts of pro-inflammatory cytokine.

**Figure 6:**
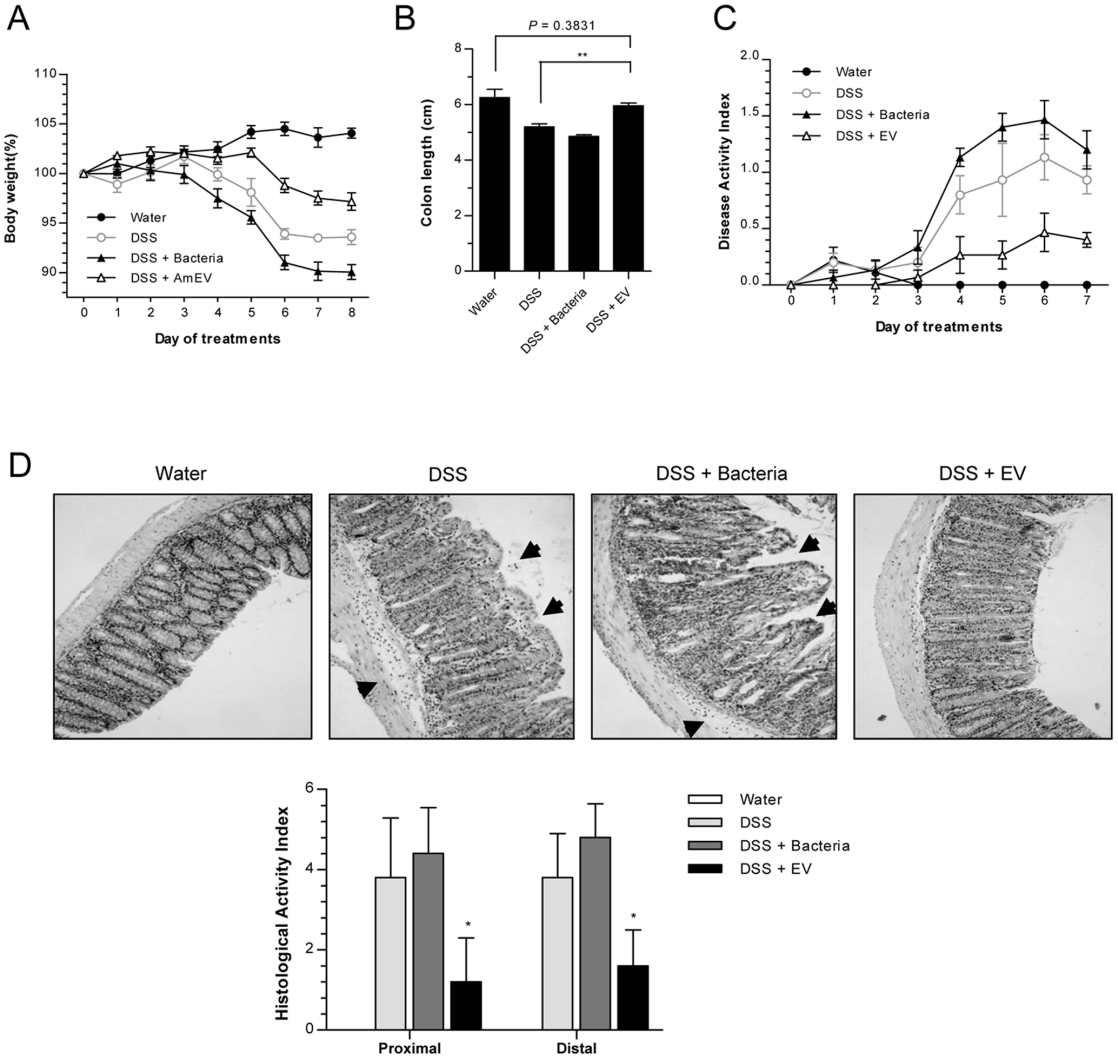
*In vivo* protective effect of *A. muciniphila*-derived EV on the development of 2% DSS-induced colitis. For all figures, mice (each group = 5) were ingested by *A. muciniphila* (Bacteria, 5.0×10^8^) or *A. muciniphila*-derived EV (Am EV, 100 µg), concomitantly with water or 2% DSS. (A) Body weight changes (%). (B) Colon lengths. **, p<0.01. (C) Changes of disease activity index, reflected by body weight, colon length, diarrhea, and stool blood. (D) Histology (upper panel) and histological activity index (lower panel) of colon. Arrows indicate injury area, and histological activity index was measured by epithelial barrier disruption and infiltration depth of inflammatory cells. *. p<0.05 vs. the DSS and DSS+Bacteria groups.

To examine the *in vivo* protective effects of *A. muciniphila*-derived EV on mice with colitis, 2% DSS was administered to female C57BL/6 mice for 5 days. 2% was administered, not the original 3%, because 3% DSS proved to be too severe. There were 4 sets of mice; mice treated only with water, treated only with 2% DSS, treated with 2% DSS and *A. muciniphila* (5×10^8^ CFU/mouse), and treated with 2% DSS and *A. muciniphila*-derived EV (AmEV, 100 mg/mouse). To analyze the severity of disease in each of these sets, body weight loss, colon length at day 8, and DAI used. Compared to the body weight loss of 2% DSS treated mice, the DSS+AmEV group lost less weight while DSS+bacteria actually showed more weight loss (**Fig. 6A**). DSS+AmEV group had a longer colon length and a lower DAI score compared to the 2% DSS group (**Fig. 6B and 6C**). Histology samples of each group showed that epithelial stability and inflammatory cell infiltration of the colon wall was ameliorated in the *A. muciniphila*-derived EV treated group (**Fig. 6D**). The comparison of body weight loss, colon length, DAI score, and histology indicated that administration of *A. muciniphila*-derived EV had a protective function that ameliorated the severity of 2% DSS-induced colitis.

## Discussion

Recently, we are flooded with evidence linking gut microbiota and intestinal immunity [13]–[15]. Research shows a causal relationship of dysbiosis of gut microbiota and IBD in genetically susceptible individuals. However, there are currently no studies involving bacteria-derived EV and its role in intestinal immunity and IBD. Our study demonstrates that bacteria EV play an important role as a mediator of gut immunity by presenting the protective characteristic of bacteria EV through our candidate *A. muciniphila*-derived EV in IBD. We are the first to use metagenomic analysis to evaluate the composition and proportional changes of gut bacteria-derived EV in the progression of colitis. Using metagenomic analysis, we showed that the proportions of gut bacteria-derived EV changes in the course of colitis induced by DSS. In particular, our studies showed an inverse relationship between *A. muciniphila*-derived EV and the severity of colitis, suggesting an important role of *A. muciniphila*-derived EV in the maintenance of intestinal homeostasis. Our studies show a compositional change in bacteria-derived EV in the course of colitis and suggest bacteria-derived EV profoundly influence host immunity and participate in human health and disease.

The principles that govern host-microbiota relationship are the focus of studies that increasingly suggest that gut microbiota-derived EV influences intestinal immunity and participates in both human health and disease [16]. With the complex mechanisms involved in the host environment and microbial community structures, it is difficult to understand this host-symbiont relationship. A myriad of reports show that gut microbiota or EV have a protective function in intestinal immunity [17]–[19]. Our present study showed that compared to the control group (2% DSS treated mice), mice treated with an oral application of 2% DSS and *A. muciniphila*-derived EV showed a decrease in body weight loss, increase in colon length, a lower DAI score, and histology that revealed a better epithelial stability and less inflammatory cell infiltration of the colon wall. Taken together, gut bacteria-derived EV play an important role in maintenance of immune homeostasis in the gut.

*A. muciniphila* is a mucin-degrader that uses mucin as nutrients [20]. Many mucolytic bacteria like *A. muciniphila* thrive in healthy human intestines; however, in CD and UC, there is a large reduction in the number of these bacteria [21]. *A. muciniphila* is already a well-known bacterium as a modulator for gut homeostasis [22]. However, in our present study the DSS+bacteria group interestingly showed an exacerbation of colitis compared to the 2% DSS control group and DSS+AmEV group. DSS+AmEV group showed a decrease in the severity of colitis while DSS+bacteria group showed an increase in the severity. As complex the interactions are between gut microbiota and host environment, it is important to consider the reciprocal nature of the regulation of the immune system and of microbial community structures. In conditions of disease, as of colitis, there is a break in the normal immunity like a disrupted gut barrier that allows abnormal passage of gut microbiota into the host system. As in our experiment, DSS treatments make the murine intestinal mucosal layer thin and make the mucosal layer permeable to infiltrate bacteria [23]–[26]. Gut microbiota like *A. muciniphila* that normally have a protective function in gut immunity, may have a harmful effect in conditions of a disrupted barrier. The role of microbiota ultimately depends on the gut environment and in conditions of colitis, application of *A. muciniphila* showed an adverse effect in the progression of DSS-induced colitis. However, administration of *A. muciniphila*-derived EV demonstrated a decrease in the severity of DSS-induced colitis. While protective microbiota in normal immune systems may have detrimental effect in disrupted host immunity, microbiota-derived EV maintains its protective factor in the disrupted host immunity. We are suggesting it is microbiota-derived EV that ultimately contains the key component in regulation of gut immunity and homeostasis.

As some bacteria are being investigated for its protective qualities in regards to the human immunity, other microbiota have generated interest in their pathogenic features that link them to certain diseases [27]. The candidate division TM7 is a recently described subgroup of Gram-positive uncultivable bacteria that inhabit a wide range of environments [28]. Previous studies have already shown an association of the TM7 bacterial division with the inflammatory pathogenesis of periodontitis [29]. More recent evidence reported a shift of TM7 bacteria in IBD patients when compared to healthy controls [30]. It is suggested that TM7 bacteria may play an important role in IBD similar to that previously described in oral inflammation. A change in the TM7 proportion was reflected also in our present metagenomic studies of gut microbiota and gut microbiota-derived EV. Compared to minor shift that occurred in bacteria composition through metagenomic analysis, we were able to clearly show that there is a far more dramatic increase of TM7-derived EV that suggests a stronger correlation between colitis and bacteria-derived EV rather than the disease and bacteria. Unfortunately, as TM7 bacterial phylum has no cultivable species, for the time being we can only suggest the role TM7-derived EV may have in the pathogenesis of IBD. Further studies will be needed to understand the mechanisms that underlie the role of bacteria-derived EV in pathogenesis of disease.

Metagenomics has emerged as a powerful tool to analyze microbial communities and to survey the different microorganisms in a specific environment [31], [32]. Metagenomic analysis has been previously done involving the microbial community of the gastrointestinal tract. We are the first to use this technology to analyze the composition of gut microbiota-derived EV. Before metagenomics, microbes were identified by taxonomic techniques that required cultivation of isolated strain *in vitro*. A majority of gut microbiota including the TM7 phylum, are uncultivable and thus metagenomics is a great leap into understanding genetic composition of the complete microbial population. We used metagenomics to survey the composition of microbiota-derived EV and the shift in proportion of microbiota-derived EV after induction of colitis by DSS. We were able to identify which microbiota-derived EV increased or decreased significantly and this provided us with a selection of candidate bacteria-derived EV.

In summary, to our knowledge we are the first use metagenomics analysis for a comprehensive evaluation of the gut microbiota-derived EV composition. Our data provides insight into the role of gut microbiota-derived EV has in regulation of intestinal immunity and homeostasis. In an environment similar to UC calibrated by DSS administration, some gut microbiota-derived EV, like TM7 bacteria, proliferated indicating a pathogenic nature and perhaps even a causal relationship with colitis. Other gut microbiota-derived EV diminished, in particular *A. muciniphila*- and *B. acidifaciens*-derived EV. *A. muciniphila*-derived EV showed to have protective effects that staggered the severity of colitis. Our study demonstrates the possibilities of stool-derived EV as a biomarker and therapeutic agents. Further investigation will lead to a more in depth understanding of the mechanisms underlying complex interactions of microbiota-derived EV and intestinal homeostasis and disease.

## Materials and Methods

### Ethics Statement

This study was carried out in strict accordance with the recommendations in the Guide for the Care and Use of Laboratory Animals of the National Institute of Health. The experimental protocols were approved by the Institutional Animal Care and Use Committee at POSTECH, Pohang, Republic of Korea (Permit Number: 2011-01-0027). All animal experiments were planned in order to minimize mice suffering.

### Mice

Specific pathogen free C57BL/6 mice were purchased from Jackson Laboratories (Bar Harbor, ME, US) and were bred in an animal laboratory at POSTECH Biotech Center. Age-and sex-matched mice were used for animal experiments.

### A mouse model of DSS-induced colitis

To generate an acute colitis mouse model, DSS (36 k-50 kD; MP Biomedicals, LLC, Illkirch, France) was added to drinking water at a concentration of 2% or 3% (weight/volume) for mice. Mice were exposed to DSS for 5 days. Healthy control animals received the drinking water only. Disease activity index (DAI) was performed in a previously published scoring system [33], [34]. The colonic tissues were processed in a paraffin cassette to stain with hematoxylin and eosin (H&E). Histological activity index (HAI) was scored in the previously described method [35]. Duplicate experiments were performed to evaluate the therapeutic effects of bacterial EV on the protection of IBD expression.

### EV isolation and characterization

EV in small intestinal fluids, stools and culture media of *A. muciniphila* were isolated by ultracentrifugation at 200,000 g for 2 h at 4°C, as the previously described method [36]. EV was characterized by TEM, NTA, and western blot. More details can be found in Text S1.

### Bacteria culture

*A. muciniphila* (ATCC BAA-835) was cultured in an anaerobic condition that was maintained to 90% N2 and 10% CO2, the method previously described [20].

### *In vitro* evaluation of EV immunogenicity

Immunogenicity of EV was evaluated in colon carcinoma cells (CT26 cells) and macrophage cell lines (Raw264.7 cells). More details can be found in Text S1.

### Metagenome sequencing

DNA was extracted from bacteria and EV in small intestinal fluids and stools. Metagenomic studies were conducted through high-throughput sequencing after amplification of the isolated DNA using common bacterial 16S rDNA. More details can be found in Text S1.

### Bioinformatics

The proportion of bacteria and bacterial EV was evaluated by phylogenetic tree generation from sequence reads. The significance of taxonomic variation was then determined by PCA.

### Statistical analysis

To confirm the difference between two groups, unpaired t-tests were performed. For the statistical analysis to several groups, an analysis of variance (ANOVA) and test of linearity were used. The statistical significance was set a priori at p<0.05.

## Supporting Information

Text S1 Supporting information.
(DOCX)
