# Supplementary material for: Extracellular Vesicles Derived from Gut Microbiota, Especially Akkermansia muciniphila, Protect the Progression of Dextran Sulfate Sodium-Induced Colitis
Source: PLoS One. 2013 Oct 24;8(10):e76520. doi: 10.1371/journal.pone.0076520 (PMC3811976; doi:10.1371/journal.pone.0076520)
Supplement: Text S1 — Supporting information. (DOCX) [file pone.0076520.s001.docx]

**Supporting information**

**Materials and Methods**

**Isolation of EV in small intestinal fluids, stools and culture media**

Stool was obtained by gathering from the mice cages and stored at -80℃ daily. Stool was dissolved in filtered PBS. Small intestinal fluids were obtained by euthanasia on day 5; small intestine was trisected and administered by filtered PBS and then fluids were collected. The isolation procedure of EV was same with stool-derived EV. To remove bacteria and other debris, 40 ml of dissolving stool was centrifuged at 10,000g for 20 min. After centrifugation, supernatants were filtered through 0.45 μm. For extraction of gut microbe-derived EV, we used sucrose density-gradient ultracentrifugation at 100,000g for 2 h at 4℃. Then EV were isolated by ultracentrifugation at 200,000 g for 2 h at 4℃ and dissolved by filtered PBS. Isolation of *A. muciniphila*-derived EV was performed by the previously described method [1].

**EV characterization**

***Transmission electron microscope (TEM)*** For the evaluation of EV shape and size, EV in filtered PBS were covered on 400-mesh copper grids to stain the sample with 2% uranyl acetate. To gain the image, we used a JEM1011 microscope (JEOL) at an accelerating voltage of 100 kV.

***Nanoparticle tracking analysis (NTA)*** Samples were loaded into the LM10 unit chamber (Nanosight, Amesbury, UK). Each sample was recorded after 30 sec and underwent Brownian motion in a 250 ml chamber through the 405-nm laser beam at 25℃. Data analysis was performed on NTA 2.3 build (Nanosight). The shutter speeds were 14.99 milliseconds. Software settings for analysis were: Detection Threshold: 5; Blur: auto; Viscosity: 0.89 cP Minimum expected particle size: auto [2].

***Western blot*** To compare the differences between daily EV, we used SDS-PAGE gel. Separating gel was 10% w/v and stacking gel was 5% w/v. We loaded 20 μg of each sample and stained the samples by using coomassie brilliant blue G-250.

***In vitro* evaluation of immune responses**

Colon carcinoma cells (CT26 cells) were plated in RPMI-1640 medium containing 5% fetal bovine serum (FBS). Macrophage cell lines in ascites (Raw264.7 cells) were coated in Dulbecco’s Modified Eagle’s Medium containing 5% fetal bovine serum. To examine the immunogenicity of EV, samples were prepared in FBS-deficient medium. The levels of an pro-inflammatory cytokine, IL-6, were quantified via ELISA by following manufacturer’s instruction (R&D systems, Minneapolis, MN, US).

**Metagenomic analysis**

***DNA extraction and emulsion-based PCR (emPCR)*** The extraction method for bacterial DNA was performed by using a stool DNA extraction kit (Bioneer Inc, Korea). Library was prepared using PCR products according to the GS FLX titanium library prep guide. Libraries were quantified using Picogreen assay (Invitrogen). The emPCR, corresponding to clonal amplification of the purified library, was carried out using the GSFLX titanium emPCR Kit (454 Life Sciences). Briefly, libraries were immobilized onto DNA capture beads. The library-beads obtained were added to a mixture of amplification mix and oil and vigorously shaken on a Tissue Lyser II (Qiagen) to create "micro-reactors" containing both amplification mix and a single bead. Emulsion was dispensed into a 96-well plate and the PCR amplification program was run according to the manufacturer's recommendations.

***Next Generation Sequencing using Roche 454 GS FLX Titanium*** Following PCR amplification, the emulsion was chemically broken and the beads carrying the amplified DNA library were recovered and washed by filtration. Positive beads were purified using the biotinylated primer A (complementary to adaptor A), which binds to streptavidin-coated magnetic beads. The DNA library beads were then separated from the magnetic beads by melting the double-stranded amplification products, leaving a population of bead-bound single-stranded template DNA fragments. The sequencing primer was then annealed to the amplified single-stranded DNA. Lastly, beads carrying amplified single-stranded DNA were counted with a Particle Counter (Beckman Coulter). Sequencing was performed on a Genome Sequencer FLX titanium (454 Life Sciences), and each sample was loaded in 1 region of a 70 mm-75 mm PicoTiter plate (454 Life Sciences) fitted with a 8-lane gasket.

***Selection of 16S rRNAs and taxonomic assignment*** By BLASTN [3], all the sequence reads were compared to EzTaxon database [4,5]. Sequence reads which has the similar sequence with more than 100 bit score and less than 1.0 E-value were admitted as partial 16S rRNA sequence. Non-16S rRNA sequence reads were less than 1%. Taxonomic assignment of the sequenced read was carried out using EzTaxon. From database, the five most similar sequences for each sequence read were found by their bit scores from BLASTN program. Among these five sequences, similarity with the sequence read was calculated by ClustalW [6], and the taxonomy of the sequence with the highest similarity was assigned to the sequence read. By the similarity, we assigned the taxonomy down to these taxonomical hierarchies; species with more than 97% similarity, genus 94%, family 90%, order 85%, class 80%, and phylum 75%.

***Phylogenetic tree generation from sequence reads*** One of the problems when dealing with metagenomics data is their huge size, which results in enormous computation power for analyses. For pairwise sequence alignment and phylogenetic tree generation in the newick tree format, clustalw2 (ver. 2.0.10) was used with ‘QUICKTREE’ option. The computer with 12 Intel 2.8 GHz CPUs and 48 GB RAM was employed for computation of ~20,000 sequence reads. For visualization of the tree, Dendroscope 3 was used [7]. Colors for each phylum were applied by manual curation. The sequences of assigned species were retrieved from NCBI GenBank by manual curation [8]. For pairwise sequence alignment and phylogenetic tree generation in the newick tree format, clustalw2 (ver. 2.0.10) was used with default options. Visualization of the tree was performed by Itol [9,10].

***Visualization and principal component analysis (PCA)*** Phylum composition was visualized with heatmap and multiple bar graph by Matlab 2011a and Excel 2010. Overall taxonomic variation between samples was statistically tested by KS-test [11]. Comparison between phylum composition was also tested by Fisher’s exact test [12]. Before PCA, data was normalized to have a mean of 0 and a standard deviation of 1 by linear normalization. PCA and two dimensional scatter plots with axis of the first and second principal component were calculated and drawn by Matlab 2011a.

**References**

1. Kim MR, Hong SW, Choi EB, Lee WH, Kim YS, et al. (2012) Staphylococcus aureus-derived extracellular vesicles induce neutrophilic pulmonary inflammation via both Th1 and Th17 cell responses. Allergy 67: 1271-1281.

2. Soo CY, Song Y, Zheng Y, Campbell EC, Riches AC, et al. (2012) Nanoparticle tracking analysis monitors microvesicle and exosome secretion from immune cells. Immunology 136: 192-197.

3. Altschul SF, Madden TL, Schaffer AA, Zhang J, Zhang Z, et al. (1997) Gapped BLAST and PSI-BLAST: a new generation of protein database search programs. Nucleic Acids Res 25: 3389-3402.

4. Kim OS, Cho YJ, Lee K, Yoon SH, Kim M, et al. (2012) Introducing EzTaxon-e: a prokaryotic 16S rRNA gene sequence database with phylotypes that represent uncultured species. Int J Syst Evol Microbiol 62: 716-721.

5. Chun J, Lee JH, Jung Y, Kim M, Kim S, et al. (2007) EzTaxon: a web-based tool for the identification of prokaryotes based on 16S ribosomal RNA gene sequences. Int J Syst Evol Microbiol 57: 2259-2261.

6. Myers EW, Miller W (1988) Optimal alignments in linear space. Comput Appl Biosci 4: 11-17.

7. Huson DH, Richter DC, Rausch C, Dezulian T, Franz M, et al. (2007) Dendroscope: An interactive viewer for large phylogenetic trees. BMC Bioinformatics 8: 460.

8. Benson D, Lipman DJ, Ostell J (1993) GenBank. Nucleic Acids Res 21: 2963-2965.

9. Letunic I, Bork P (2011) Interactive Tree Of Life v2: online annotation and display of phylogenetic trees made easy. Nucleic Acids Res 39: W475-478.

10. Letunic I, Bork P (2007) Interactive Tree Of Life (iTOL): an online tool for phylogenetic tree display and annotation. Bioinformatics 23: 127-128.

11. Van Bockstaele F, Janssens A, Piette A, Callewaert F, Pede V, et al. (2006) Kolmogorov-Smirnov statistical test for analysis of ZAP-70 expression in B-CLL, compared with quantitative PCR and IgV(H) mutation status. Cytometry B Clin Cytom 70: 302-308.

12. Fisher RA (1954) Statistical methods for research workers. Edinburgh: Oliver and Boyd. 356p.
